# Short-term evolocumab-induced tendon xanthomas regression in an elderly patient with homozygous familial hypercholesterolemia

**DOI:** 10.1007/s11739-022-03106-6

**Published:** 2022-10-06

**Authors:** Arrigo F. G. Cicero, Federica Fogacci, Alessio Bragagni, Claudio Borghi

**Affiliations:** 1 IRCCS Azienda Ospedaliero-Universitaria di Bologna, Bologna, Italy; 2 Hypertension and Cardiovascular Risk Factors Research Center, Medical and Surgical Sciences Department, Sant’Orsola-Malpighi University Hospital, 40138 Bologna, Italy; 3 Medical and Surgical Sciences Department, Alma Mater Studiorum University of Bologna, Bologna, Italy

Homozygous Familial Hypercholesterolemia (HoFH) is a rare inherited disorder affecting 1 in 160,000 to 1 in 300,000 individuals and resulting in extremely elevated low-density lipoprotein cholesterol (LDL-C) levels and premature atherosclerotic cardiovascular disease (ASCVD) [1]. Manifestations of ASCVD most notably include fatal and non-fatal myocardial infarction (MI) and occlusive vascular disease requiring surgical or percutaneous revascularization. Deposits of cholesterol in the skin or tendons, or both, called xanthomas, are the hallmark of the disease [1].

Below, we present a patient case of tendon xanthomas regression after short-term treatment with proprotein convertase subtilisin/kexin type 9 (PCSK9) inhibitor evolocumab.

A 72-year-old heavy-smoking Caucasian men with multidistrict atherosclerotic disease presented to Lipid Clinic because of consistently high levels of LDL-C (272 mg/dL) despite intensive lipid-lowering therapy (LLT). He took every day 40 mg rosuvastatin, 10 mg ezetimibe, 100 mg acetyl salicylic acid, 40 mg pantoprazole, 300 mg allopurinol, 2.5 mg bisoprolol and 25 mg furosemide. However, he reported that he had never taken any medications before experiencing acute coronary syndrome and undergoing percutaneous transluminal coronary angioplasty (PTCA) and stenting, 5 years before. Subsequently, he had also been treated with carotid endarterectomy (CEA) for severe asymptomatic carotid stenosis.

Physical examination was positive for *arcus cornealis* and bulky tendon xanthomas in the fingers, elbows and ankles, for which the patient had previously referred to an orthopedic clinic because causing movement limitation. Based on radiographic evidence, the orthopedic specialist had diagnosed gouty tophi and surgically removed the lesions without requesting any histological confirmation. After surgery, the patient had been started on allopurinol and advised to limit consumption of purine rich food. Serum uric acid (SUA) concentrations quickly reduced to < 2 mg/dL, but all nodules obviously relapsed after few weeks from surgery. However, the patient reported that he was not surprised, because he “had always had them” and stated that his brother and sister had also had them.

**Fig. 1:**
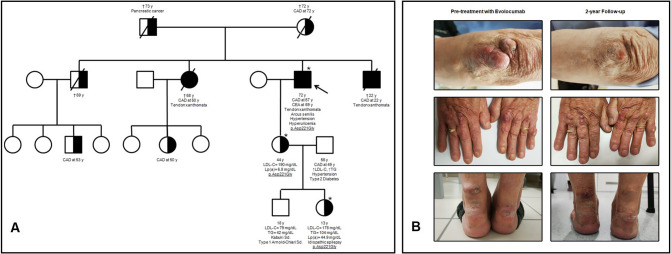
Family tree (**A**). Evolocumab-induced tendon xanthomas regression in elbow (above), fingers (in the middle) and ankles (below) (**B**)

Considering the remarkable family history for ASCVD (Fig. 1A) and the very high LDL-C despite maximal standard LLT, the patient was clinically diagnosed with familial hypercholesterolemia (FH). Then, the sequence of LDLR gene revealed that he was homozygous for a single-nucleotide substitution (c.662A > G) in exon 4, resulting in glycine for aspartic acid conversion at position 221 (p.Asp221Gly) of LDL receptor. The further mutation screening of living relatives (performed by directly sequencing of exon 4) revealed that patient’s daughter and granddaughter were heterozygous for the same mutation (Fig. 1A), that in silico analysis predicted to be “pathogenic”.

After being adequately informed of the benefits and risks, the patient refused to be treated with both lipoprotein apheresis and lomitapide, and he was started on subcutaneous administration of 420 mg of evolocumab every 2 weeks. The LLT was effective and safe (Table 1). Tendon xanthomas regression in fingers, elbows and ankles began 6 months after starting treatment with iPCSK9 evolocumab and became more evident at 2-year follow-up, with the patient dropping two shoe sizes for the thinning of the Achilles tendons (Fig. 1B). Patient’s self-perceived quality of life improved too.

**Table 1:** Evolocumab-related changes in laboratory and hemodynamic collected variables

	Baseline	2-year follow-up
Lipid-lowering treatment	40 mg rosuvastatin and ezetimibe	40 mg rosuvastatin and ezetimibe + 420 mg evolocumab b.i.m
Laboratory parameters		
Total cholesterol (mg/dL)	340	288
HDL-cholesterol (mg/dL)	26	45
LDL-cholesterol (mg/dL)	272	220
Triglycerides (mg/dL)	132	117
Apolipoprotein B-100 (mg/dL)	177	135
Lipoprotein(a) (mg/dL)	1.9	1.5
Fasting plasma glucose (mg/dL)e	93	115
Uric acid (mg/dL)	2.5	6.5
eGFR (ml/min)	80	90
Creatinine phosphokinase (U/L)	60	152
AST (U/L)	26	33
ALT (U/L)	30	28
Gamma-GT (U/L)	23	48
Hemodynamic parameters		
Heart rate (bpm)	69	79
Systolic blood pressure (mmHg)	163	136
Diastolic blood pressure (mmHg)	64	58
Pulse pressure (mmHg)	99	78
Mean arterial pressure (mmHg)	111	91
Aortic systolic blood pressure (mmHg)	162	136
Aortic diastolic blood pressure (mmHg)	64	60
Aortic pulse pressure (mmHg)	98	76
Augmentation pressure (mmHg)	28	19
Augmentation index (%)	29	24
Cardiac output (L/min)	13.13	9.87
Peripheral resistance (PRU)	0.50	0.55
Stroke volume (mL)	191	125
Pulse Wave velocity (m/s)	10.7	10.3
Ankle-brachial index—right	0.51	0.81
Ankle-brachial index—left	0.98	1.23

*ALT* Alanine transaminases, *AST* Aspartate transaminases, *b.i.m.* bis in month, *eGFR* estimated glomerular filtration rate, *HDL* High-density lipoprotein, *LDL* Low-density lipoprotein

According to previously published data, individuals affected by HoFH usually develop ASCVD early in life. Not-pharmacologically treated individuals with HoFH suffer from MI as young as 3 years of age, and most patients experience fatal events prior to the age of 30 even if undergoing LLT [2]. However, reaching an advanced age without cardiovascular complications should not be considered an exclusion criterion neither for the diagnosis of HoFH nor for physician’s decision to start an intensive LLT, as the patient case shows. Actually, treatment with PCSK9i evolocumab was both effective and safe for our patient, with multiple implications. In effect, previous evidence shows that presence of xanthomas increases the risk of ASCVD in patients with FH by as much as threefold, implying that xanthomas and atherosclerosis may share a certain etiology [3]. Then, it is likely that tendon xanthomas regression corresponds to a reduced ASCVD risk in our patient, who did not experience neither other ASCVD events nor disease progression. Moreover, tendon xanthomas regression has been rarely observed in general and never observed in elderly patients with HoFH. In particular, our observations are unexpected since xanthomas regression resulted from a non-maximal reduction in LDL-C, and after the previous surgical lesions, removal is likely to have caused fibroblast deposition and scar tissue in the tendons. Finally, patient’s self-perceived quality of life improved after tendon xanthomas regression, and this is definitely crucial for optimizing patients’ adherence to LLT [4]. Of course, before any inference, further observations are needed to confirm ours.
